# Revisiting Triple Therapy for HCC: Progress in Survival Outcomes in Patients with Hepatic Vein and/or IVC Tumor Thrombus

**DOI:** 10.1007/s00270-025-03967-2

**Published:** 2025-01-27

**Authors:** Elif Can

**Affiliations:** Department of Diagnostic and Interventional Radiology, Faculty of Medicine, Medical Center – University of Freiburg, University of Freiburg, Hugstetter Str. 55, 79106 Freiburg, Germany

The recent study by Lin and colleagues on triple therapy—a combination of transarterial chemoembolization (TACE), Lenvatinib, and PD-1 inhibitors—provides a potentially important development for patients with hepatic vein and inferior vena cava tumor thrombus (HVTT/IVCTT), a particularly aggressive manifestation of HCC that has traditionally been associated with very poor prognosis [1, 2]. Patients treated with TACE alone had a median overall survival (OS) of just 8.3 months with dual therapy (TACE and Lenvatinib) showing a modest improvement, with an OS of 11.2 months. However, the addition of PD-1 inhibitors in the triple-therapy regimen did result in a further increase, with the median OS reaching 16.2 months [1].

While these improvements represent a notable shift from previous survival expectations, it is important to frame these changes as a relative advance in a patient population that has faced dismal survival outcomes. However, these survival gains should be understood within the context of a palliative treatment approach, rather than a curative one. Previous studies on triple therapy for HCC have generally indicated incremental rather than transformative progress [3, 4]. While the current study does suggest some benefit, it is important to temper expectations. TACE works by blocking the blood supply to the tumor and inducing ischemic necrosis, while Lenvatinib, a tyrosine kinase inhibitor, targets angiogenesis [3, 5]. PD-1 inhibitors, the key component of this regimen, unleash the immune system by removing the inhibition on T-cell activation [4, 6]. These therapies may act synergistically; their clinical benefits remain modest and must be interpreted cautiously. The higher incidence of treatment-related adverse events (TRAEs), including fatigue and skin rash, was generally manageable; however, the balance between survival extension and potential toxicity requires careful evaluation, particularly in patients with advanced disease [7].

The findings of this study hold important implications for tumor boards, prompting a reassessment of treatment strategies for HVTT and IVCTT. The results presented by Lin and colleagues suggest that more aggressive therapeutic approaches may be warranted, provided the survival benefits are balanced against the potential toxicity. Careful patient selection is essential, prioritizing individuals most likely to tolerate and derive meaningful benefit from intensive treatments.

While these results suggest some improvement, larger, prospective randomized clinical trials are necessary to confirm the safety and efficacy of triple therapy. Additionally, biomarkers to identify which patients are most likely to benefit from this approach would be essential. Emerging tools like artificial intelligence and radiomics hold significant potential for refining patient selection and guiding clinical decision-making with precision.

Overall, while the findings represent a step forward, the challenges in treating vascular invasion in HCC remain significant. Triple therapy shows potential in improving survival outcomes for this high-risk population, but further research is needed to confirm its true impact. Moving forward, the focus should remain on validating these findings through further research and ensuring equitable access for eligible patients.

Last but not least, given the ongoing debates regarding the future role of TACE in HCC therapy, recent evidence highlights its continued relevance, particularly as part of multimodal strategies, serving as a cornerstone for improving both local tumor control and overall survival.

## References

[1] Lin LW, Nian YX, Lin X, Ke K, Yang WZ, Lin JQ, et al. Efficacy and safety of transarterial chemoembolization combined with lenvatinib plus programmed death-1 inhibitor for hepatocellular carcinoma with the hepatic vein and/or inferior vena cava tumor thrombus. Cardiovasc Intervent Radiol. (2024). 10.1007/s00270-024-03919-2 39658748

[2] Alrashidi I, Chu HH, Kim JH, Shim JH, Yoon SM, Kim PH, et al. Combined chemoembolization and radiotherapy versus chemoembolization alone for hepatocellular carcinoma invading the hepatic vein or inferior vena cava. Cardiovasc Intervent Radiol. 2021;44(7):1060–9. 33745071 10.1007/s00270-021-02815-3

[3] Yuanren G, Yin L, Liu R, Cui Y. The treatment of transarterial chemoembolization/hepatic arterial infusion chemotherapy combined with lenvatinib and PD-1 inhibitor is effective against hepatocellular carcinoma with portal vein tumor thrombus: a systematic review. Front Oncol. 2023;13:1054072. 36969065 10.3389/fonc.2023.1054072 PMC10034403

[4] Chen Y, Jia L, Li Y, Cui W, Wang J, Zhang C, et al. Clinical effectiveness and safety of transarterial chemoembolization: hepatic artery infusion chemotherapy plus tyrosine kinase inhibitors with or without programmed cell death protein-1 inhibitors for unresectable hepatocellular carcinoma-a retrospective study. Ann Surg Oncol. 2024;31(12):7860–9. 39090499 10.1245/s10434-024-15933-2

[5] Al-Salama ZT, Syed YY, Scott LJ. Lenvatinib: a review in hepatocellular carcinoma. Drugs. 2019;79(6):665–74. 30993651 10.1007/s40265-019-01116-x

[6] Chen S, Xu B, Wu Z, Wang P, Yu W, Liu Z, et al. Pembrolizumab plus lenvatinib with or without hepatic arterial infusion chemotherapy in selected populations of patients with treatment-naive unresectable hepatocellular carcinoma exhibiting PD-L1 staining: a multicenter retrospective study. BMC Cancer. 2021;21(1):1126. 34670506 10.1186/s12885-021-08858-6 PMC8527794

[7] easloffice@easloffice.eu EAftSotLEa, Liver EAftSot. EASL Clinical practice guidelines on the management of hepatocellular carcinoma. J Hepatol. (2024).

